# Molecular docking analysis of curcumin analogues with COX-2

**DOI:** 10.6026/97320630013356

**Published:** 2017-11-30

**Authors:** Mario Rowan Sohilait, Harno Dwi Pranowo, Winarto Haryadi

**Affiliations:** 1 Department of Chemistry, Faculty of Mathematics and Natural Sciences, Universitas Pattimura, Jl. Ir. M. Putuhena, Kampus Poka, Ambon 97233, Indonesia; 2 Department of Chemistry, Faculty of Mathematics and Natural Sciences, Universitas Gadjah Mada, Jl. Sekip Utara, Yogyakarta 55281, Indonesia

**Keywords:** curcumin analogues, molecular docking, Autodock

## Abstract

Curcumin analogues were evaluated for COX-2 inhibitory as anti-inflammatory activities. The designed analogues significantly enhance COX-2 selectivity. The three compounds could dock into the active site of COX-2 successfully. The binding energies of -8.2, - 7.6 and -7.5 kcal/mol were obtained for three analogues of curcumin respectively. Molecular docking study revealed the binding orientations of curcumin analogues in the active sites of COX-2 towards the design of potent inhibitors.

## Background

The target for these anti-inflammatory drugs is cyclooxygenase (COX), a rate-limiting enzyme involved in the conversion of arachidonic acid into inflammatory prostaglandins. The two isozymes of COX involved in prostaglandin biosynthesis are COX-1 and COX-2. COX-1 is known as a housekeeping enzyme and constitutively expressed in all tissues, while COX-2 is constitutively expressed only in kidney, brain and ovaries. COX-2 is increasingly expressed during inflammatory conditions by proinflammatory molecules such as IL-1, TNF-α, LPS [1, 2, 3, 4] and its expression is absent or low in healthy individuals [5, 6]. Although COX-2 inhibitors are widely prescribed anti-inflammatory agents, conversely several important side effects have been associated with the simultaneous inhibition of COX-1 activity [7, 8, 9]. Therefore, the development of compounds that would inhibit COX-2 almost exclusively is an important target in order to reduce adverse side effects during non-steroidal antiinflammatory treatment, thus improving therapeutic benefits.

Although the genes of both isoforms are different, COX-1 and COX-2 have similar structures and catalytic activities. The amino acid sequences for the substrate binding and catalytic sites are almost identical, but COX-2 has valine substituted for isoleucine at positions 434 and 523 [10, 11]. Valine is smaller than isoleucine by a methyl group. These substitutions result in a larger and more flexible substrate channel and a secondary internal pocket off the inhibitor-binding site of COX-2, which is not observed in COX-1.

Curcumin is found as a major pigment in the Indian spice turmeric (C. longa, Zingiberaceae). The rhizome of the C. longa has been used in indigenous medicine for the treatment of inflammatory disorders and its medicinal activity has been known since ancient times. Curcumin is reported to have antiinflammatory, antioxidant and anticancer properties [12]. From the literature it was found that curcumin was investigated for COX inhibitory activity using bovine seminal vesicles, microsomes and cytosol from homogenates of mouse epidermis showed IC50 value of 2 μM [13], 52 μM [14], and 5-10 μM [15], respectively.

Pharmachophore modification of the dienone functional group curcumin into monoketone and side chain of aromatic ring with symmetrical or asymmetrical substituents has been might give better activity and stability than the parent compound [16, 17, 18]. Robinson, et al. has proven that the change of β-diketone on the structure into α, β-unsaturated ketone did not change the activity of the curcumin analogue to inhibit the cancer cell. Even, in several cases such compound gave better activities than the curcumin itself [19].

Molecular docking is an efficient tool to get an insight into ligand-receptor interactions. All molecular docking calculations were performed on AutoDock software. The AutoDock Tools (ADT) graphical user interface was used to calculate Kollman charges for the protein and to add polar hydrogen. Molecular docking is a computational procedure that attempts to predict non-covalent binding of macromolecules or, more frequently, of a macromolecule (receptor) and a small molecule (ligand) efficiently, starting with their unbound structures, structures obtained from MD simulations, or homology modeling, etc. The goal is to predict the bound conformations and the binding affinity. In the present study, we describe binding properties of 15 curcumin analogues to the 6COX subdomains of COX-2, using molecular docking studies.

## Methodology

### Softwares Used

The ligand preparation done by using ACD/ChemSketch 12.01 (Advanced Chemistry Development, Inc), geometries were optimized using Hyperchem 8.0.3 and for protein preparation Wizard of AutoDock tools 1.5.6 are used. Molecular docking calculation has done by AutoDock tools 1.5.6 and MGL tools 1.5.6 packages (The Scripps Research Institute, Molecular Graphics Laboratory, 10550 North Torrey Pines Road, CA, 92037).

### Docking Procedure

#### Protein Preparation

Three-dimensional coordinates COX-2 (pdb code 6-COX) were retrieved from Brookhaven Protein Data Bank. The pdb file was submitted to "Build/check/repair model" and "Prepare PDB file for docking programs" modules where missing side chains were modeled in, a small regularization was performed, water positions and symmetry were corrected, and hydrogen were added. Only chain A of the repaired pdb file was evaluated and passed to AutodockTools (ADT ver.1.5.6) for pdbqt file preparation. Thus, water molecules and non-standard residues were removed, only polar hydrogen was maintained, and Gasteiger charges were computed for protein atoms by ADT.

#### Ligands Preparation

All the molecules were constructed with ChemSketch-12.01 program and these geometries were optimized using the Austin Model 1 to the corresponding mol2 file that was submitted to ADT for pdbqt file preparation and docking with AutoDock4. The geometry of built compound was optimized, partial charges were also calculated, and saved as mol2 files that was passed, as usual, to ADT for pdbqt file preparation.

#### Docking Procedure

Autodock4 (ver. 4.2.6) [20, 21] was employed for docking simulations. Lamarckian genetic algorithm with local search (GALS) was used as search engine, with a total of 100 runs. The region of interest, used by Autodock4 for docking runs and by Autogrid4 for affinity grid maps preparation, was defined in such a way to comprise the whole catalytic binding site using a grid of 40 x 40 x 40 points with a grid space of 0.375 Å, centers of grid box: x = 23.049; y = 23.526; z = 46.984. Cluster analysis was performed on the docked results using an RMS tolerance of 2.0 Å. Finally; the more energetically favorable cluster poses were evaluated by using Python Molecule Viewer (PMV ver.1.5.6) and PyMOL ver.1.1.7 (DeLano Scientific LLC).

The level of COX-2 inhibitory and anti-inflammatory activities of 15 curcumin analogues Table 1, prompted us to perform molecular docking studies to understand the ligand-protein interactions and COX-2 selectivity in detail. All the calculations were performed using Autodock Tools (ADT) ver.1.5.6. The crystal structures of COX-2 enzymes complexes with SC-558 [6COX.pdb] were used for docking. Extracting co-crystallized inhibitor from the protein and then docking the same tested the docking protocol. The docking protocol predicted the same conformation as was present in the crystal structure with RMSD value well within the allowed range of 2 Å [22].

The ADT program is an automated docking program, was used to dock compounds curcumin analogues on the active sites of COX-2 enzymes. For each compound the most stable docking model was selected according to the best scored conformation predicted by the Autodock scoring function. The complexes were energy-minimized with an Austin model 1 force field till the gradient convergence 0.01 kcal/mol was reached.

**Figure 1:**
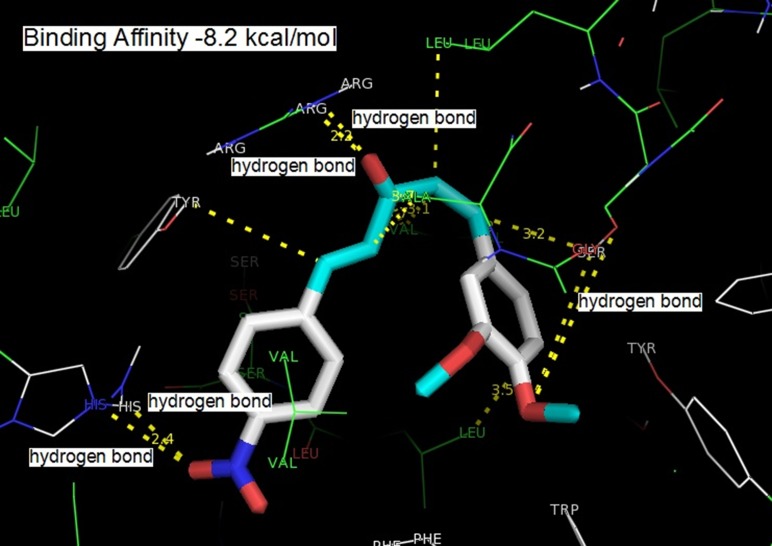
Binding of 1-(3,4-dimethoxyphenyl)-5-(4-nitrophenyl) penta-1, 4-dien-3-one into the active site of COX-2

The three compounds could dock into the active site of COX-2 successfully. The binding energies of -7.5, -8.2 and -7.6 kcal/mol were obtained for 1-(1,3-benzodioxol-5-yl)-5-(4-hydroxy-3- methoxyphenyl) penta-1, 4-dien-3-one, 1-(3,4-dimethoxyphenyl)- 5-(4-nitrophenyl) penta-1, 4-dien-3-one and 1-(4-hydroxy-3- methoxyphenyl)-5-(4-methoxyphenyl) penta-1, 4-dien-3-one, respectively. The lower interaction energy observed for 1-(3,4- dimethoxyphenyl)-5-(4-nitrophenyl) penta-1, 4-dien-3-one rationalizes the tighter binding of curcumin analogue (Figure 1) into the COX-2 active site than that of the other two compounds. The tight binding can be explained in terms of extra hydrogen bonding with monoketone C=O and Arg 120. All the three compounds were involved in the hydrogen bonding with a residue Ser 530. The hydrogen bonding distance between one of the methoxy group of curcumin with OH of Ser 530 was found to be 3.6 Å (o...O), 3.3 Å (O...H). Active site amino acid residues Ser 530, Gly 526, Met 522, Tyr 385 and Ala 526 surrounded one of the phenyl rings of curcumin. Residues Tyr 355, Ala 527, Ser 353, Leu 531, and Val 352 surrounded the pentanoid part. Phe 518, His 90, Val 523, Ser 353 and Leu 351 surrounded the second phenyl ring. A similar trend was observed for 1-(3,4-dimethoxyphenyl)-5-(4- nitrophenyl) penta-1, 4-dien-3-one and 1-(4-hydroxy-3- methoxyphenyl)-5-(4-methoxyphenyl) penta-1, 4-dien-3-one complexes.

**Figure 2:**
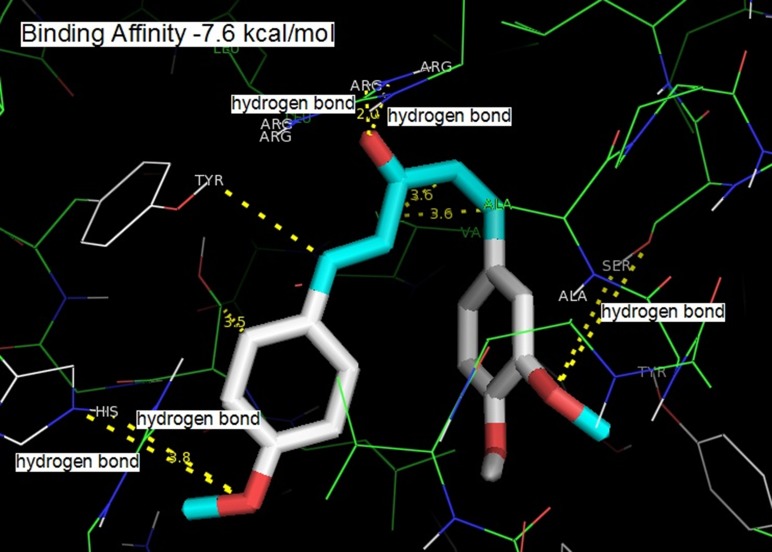
Binding of 1-(4-hydroxy-3-methoxyphenyl)-5-(4- methoxyphenyl) penta-1, 4-dien-3-one into the active site of COX-2

**Figure 3:**
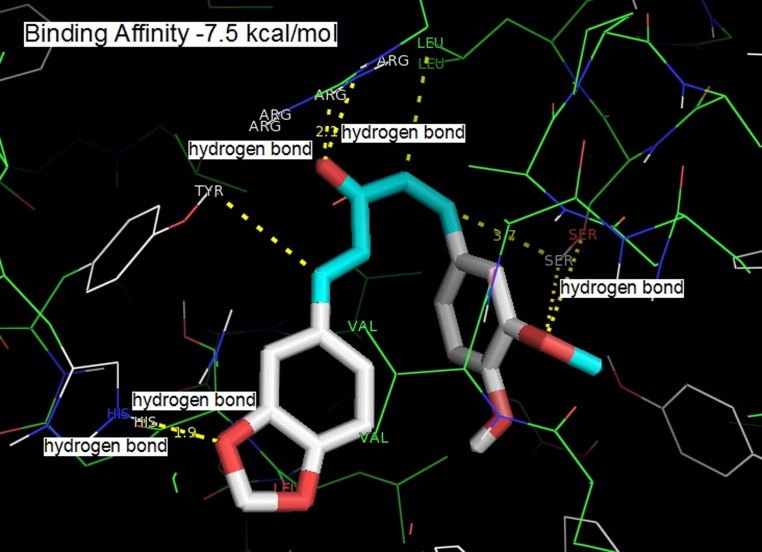
Binding of 1-(1,3-benzodioxol-5-yl)-5-(4-hydroxy-3-methoxyphenyl) penta-1, 4-dien-3-one into the active site of COX-2

The hydrogen bonding between OH of the second phenyl ring and His 90 (3.2 Å, O...N, 2.4 Å, O...H-N; 4.5 Å, O...N, 3.8 Å, O...H- N; 2.7 Å, O...N, 1.9 Å, O...H-N were obtained for 1-(3,4- dimethoxyphenyl)-5-(4-nitrophenyl) penta-1, 4-dien-3-one, 1-(4- hydroxy-3-methoxyphenyl)-5-(4-methoxyphenyl) penta-1, 4-dien- 3-one and 1-(1,3-benzodioxol-5-yl)-5-(4-hydroxy-3- methoxyphenyl) penta-1, 4-dien-3-one, respectively) was observed. C=O of the monoketone was involved in hydrogen bonding interaction with Arg 120 (2.7 Å, N...O, 2.2 Å, O...H) (Figure 1), (2.8 Å, N...O, 2.0 Å, O...H) (Figure 2), (3.0 Å, N...O, 2.1 Å, O...H) (Figure 3). The curcumin analogue 1-(1,3-benzodioxol-5-yl)-5-(4- hydroxy-3-methoxyphenyl) penta-1, 4-dien-3-one orients in a similar fashion to that of 1-(3,4-dimethoxyphenyl)-5-(4-nitrophenyl) penta-1, 4-dien-3-one and 1-(4-hydroxy-3- methoxyphenyl)-5-(4-methoxyphenyl) penta-1, 4-dien-3-one. However, only one hydrogen bond was observed between the methoxy group and OH of Ser 530 (3.6 Å, O...O, 3.8 Å (O...H) (Figure 3). Based on this study, there are three curcumin analogues showed significant inhibition of the enzyme COX-2. It is clear that this compound has the potential to inhibit COX enzymes, however, they need to be confirmed from the biological evaluation and in vitro testing.

## Conclusion

Three curcumin analogues were investigated for COX-2 inhibitory activities. Pharmachophore modification of the dienone functional group into monoketone and side chain of aromatic rings with symmetrical or asymmetrical substituents give better activity and stability than the parent compound. Molecular docking studies further helps in understanding the various interactions between the ligands and enzyme active sites in detail and thereby helps to design novel potent inhibitors.

## Figures and Tables

**Table 1:** Molecular docking of 15 curcumin analogues

IUPAC names	Binding Affinity (Kcal/mol)	RMSD (Å)
1-phenylsulfonamide-3-trifluoromethyl-5-parabromophenylpyrazole	-8.6	1.514
1-(1,3-benzodioxol-5-yl)-5-(4-hydroxy-3-methoxyphenyl) penta-1, 4-dien-3-one	-7.5	1.6
1-(1,3-benzodioxol-5-yl)-5-(3,4-dimethoxyphenyl) penta-1, 4-dien-3-one	-7	1.58
1-(1,3-benzodioxol-5-yl)-5-(4-methoxyphenyl) penta-1, 4-dien-3-one	-6.9	1.504
1-(1,3-benzodioxol-5-yl)-5-[4-(dimethylamino) phenyl] penta-1, 4-dien-3-one	-6.4	1.67
1-(1,3-benzodioxol-5-yl)-5-(4-nitrophenyl) penta-1, 4-dien-3-one	-7	1.635
1-(3,4-dimethoxyphenyl)-5-(4-hydroxy-3-methoxyphenyl) penta-1, 4-dien-3-one	-7.2	1.586
1-(3,4-dimethoxyphenyl)-5-(4-methoxyphenyl) penta-1, 4-dien-3-one	-6.5	1.608
1-(3,4-dimethoxyphenyl)-5-[4-(dimethylamino) phenyl] penta-1, 4-dien-3-one	-6.2	1.533
1-(3,4-dimethoxyphenyl)-5-(4-nitrophenyl) penta-1, 4-dien-3-one	-8.2	1.41
1-(4-hydroxy-3-methoxyphenyl)-5-(4-methoxyphenyl) penta-1, 4-dien-3-one	-7.6	1.509
1-[4-(dimethylamino) phenyl]-5-(4-hydroxy-3-methoxyphenyl) penta-1, 4-dien-3-one	-6.7	1.709
1-(4-hydroxy-3-methoxyphenyl)-5-(4-nitrophenyl) penta-1, 4-dien-3-one	-7.2	1.62
1,5-bis (4-methoxyphenyl) penta-1, 4-dien-3-one	-6.1	1.781
1,5-bis [4-(dimethylamino) phenyl] penta-1, 4-dien-3-one	-7	1.693
1,5-bis (4-nitrophenyl) penta-1, 4-dien-3-one	-6.8	1.705
